# Veterans' experiences of somatic experiencing and prolonged exposure therapies for post‐traumatic stress disorder: A qualitative analysis

**DOI:** 10.1111/papt.12570

**Published:** 2025-01-14

**Authors:** Anna Harwood‐Gross, Shir Elias, Karen Lerner, Nitsa Nacasch, Cathy Lawi, Danny Brom, Adi Barak

**Affiliations:** ^1^ Metiv Israel Psychotrauma Center Herzog Medical Center Jerusalem Israel; ^2^ Leiden University Medical Center Leiden University Leiden Netherlands; ^3^ Psychologist in private practice Herzliya Israel; ^4^ Sheba Tel ha Shomer Hospital Ramat Gan Israel; ^5^ Emotion Aid Raanana Israel; ^6^ The Louis & Gabi Weisfeld School of Social Work Bar‐Ilan University Ramat Gan Israel

**Keywords:** cognitive behavioural therapy, body, evidence based practice, military, psychotherapy, qualitative, somatic, trauma

## Abstract

**Purpose:**

Despite the proliferation of research into evidence based treatment for military PTSD there is little evidence for treatment assignment criterion and military based PTSD still demonstrates low remission rates.

**Method:**

Thirty participants in a randomized control trial comparing Prolonged Exposure (PE) and Somatic Experiencing (SE) were interviewed on their experiences in therapy and their responses assessed using a descriptive phenomenological analysis approach to delineate the central tenets of the two therapeutic approaches.

**Results:**

Results indicated that participants from both therapies covered themes of the experience of change, the experience of the therapeutic relationship and the therapeutic process. Within these themes, SE and PE participants reported both similar experiences, such as the predominance of physiological or bodily experiences and also described nuanced differences, specifically pertaining to therapy characteristics. SE participants described the process in terms of learning a language, applicable to alternative scenarios and PE participants described the process in terms of conquering exposures in order to achieve respite from symptoms.

**Conclusion:**

The current findings have relevance in terms of presenting the key elements of the distinct trauma therapies and determining treatment appropriateness based on desired outcomes. They highlight the commonalities and differences between the patient experience in PE and SE, specifically the relevance of the bodily response, treatment expertise and therapist characteristics in both treatments. Understanding the unique elements of PE, a gold‐standard PTSD treatment and SE, a novel somatic‐based psychotherapy, will allow for better treatment preparation for participants and potentially aid treatment assignment.

## INTRODUCTION

Post‐traumatic stress disorder (PTSD) treatments for veterans demonstrate poor levels of remission, around a third (Forbes et al., 2019; Steenkamp et al., 2015) and the most effective, gold‐standard treatments, such as Prolonged Exposure (PE) have high rates of attrition (55% in a recent study; Schnurr et al., 2022). Whilst veteran associations typically offer short, evidence based treatments such as Prolonged Exposure (PE), Cognitive Processing Therapy (CPT) and Eye movement desensitization and reprocessing (EMDR), there has been little success in delineating successful treatment assignment procedures, or in short, who is likely to benefit from which treatment. Additionally, in recent years there has been an increase in new treatments for PTSD involving somatic and psychedelic approaches which offer a new approach to PTSD treatment though still have a limited evidence base (Bisson & Olff, 2021; Burback et al., 2023).

When determining treatment assignment, practitioners refer to patient related factors such as willingness to discuss trauma, symptom severity and coping skills (Finley et al., 2019) though there is little evidence to support these practices and these decisions may influence why, even amongst trained practitioners, evidence based practices (EBPs) make up less than half their caseload (Finley et al., 2015).

Recently, there has been an enhanced effort to structure PTSD stage or severity in order to refer to the most appropriate treatments (Burback et al., 2023; McFarlane et al., 2017) and also to consider factors which may be pertinent to specific treatment success (Stojek et al., 2018; van Toorenburg et al., 2020). Additionally, patients' lived experiences of PTSD therapies and attrition are getting wider research attention through the implementation of qualitative or mixed method research strategies (Doran et al., 2021; Hundt et al., 2020; Meis et al., 2022; Sherrill et al., 2022).

Understanding participants' experiences of PTSD therapies may help delineate the central tenants of the therapeutic experience during different therapies, not captured by quantitative research. This may aid preparatory work in anticipation of trauma focused therapies by clearly delineating differential factors between treatments and enabling a more educated decision by both potential patients and referring therapists. Additionally, many intensive and massed treatment approaches for PTSD use combinations of psychological and functional treatments (Wright et al., 2022) and the integration of somatic, EMDR or psychedelic approaches with cognitive behavioural therapy (CBT) treatments has become more common, especially with complex PTSD presentations (Bisson & Olff, 2021; Bongaerts et al., 2022; Lahad et al., 2016; Voorendonk et al., 2020). It is commonplace for therapists to be trained in multiple methods and rather than specific competencies, the therapeutic alliance is frequently defined as a central predictor of treatment success indicating that the central tenets of individual treatment approaches have still not been delineated (Steil et al., 2023).

Qualitative research can offer an alternative to theory‐testing research, specifically when there are still gulfs in the knowledge of what contributes to the low success rates of PTSD therapies. For example, whilst it is predominantly the clinician's perspective which drives quantitative research, Meis and colleagues highlighted the difference between clinicians' views of causation of poor adherence versus patients' perspectives (Meis et al., 2022). Until now, qualitative research has focused on singular treatment modalities for PTSD including, CBT (PE or CPT) (Hundt et al., 2020; Thompson‐Hollands et al., 2019), EMDR (Whitehouse, 2021) and psychedelic (Barone et al., 2019) therapies but the exploration of the experience of patient groups assigned concurrently to different trauma therapies has been weekly explored.

The current study was designed to explore the participants' experience of partaking in a randomized control trial for two therapies for the treatment of PTSD; PE and Somatic Experiencing (SE).

PE is based on the Emotional Processing Theory, developed by (Foa & Kozak, 1986). According to the theory, post‐traumatic stress disorder results from the development of a pathological emotional‐cognitive memory structure that developed following a traumatic event. This structure is characterized by representations of excessive trauma‐related stimuli, representations of the person's reactions during the event and the pathological meanings of those stimuli and reactions. In survivors who suffer from PTSD, large number of trauma‐related stimuli are inadvertently associated to danger (for example, darkness, being alone, crowded places) and the responses during and after the trauma are associated with basic dysfunctional cognitions that underlie the development and maintenance of PTSD, that is the world is completely dangerous and thoughts of incompetence (Foa et al., 1999). These erroneous cognitions are reflected in avoidance (i.e. avoiding traumatic reminders by staying alone at home or not going out in the dark or to crowded places) and this avoidance subsequently prevents change of the erroneous cognition that the world is completely dangerous. Moreover, the perception of the world as a dangerous place is expressed in symptoms of hypervigilance, hyperarousal and physical reactions in response to trauma reminders.

Memory representations of the trauma and subsequent negative interpretation of their emotional, behavioural and cognitive reactions during and after the event can also lead to negative feelings like shame, guilt, anger, fear and sadness and as with previous avoidant behaviours, the avoidance of trauma‐related thoughts and feelings does not offer the opportunity to incorporate disconfirming information and processes negative responses. It is thus posited that persistent emotional disturbances such as PTSD following a traumatic event may indicate inadequate processing of the trauma memory and thus the recovery process involves the organizing and streamlining of the memory (Foa et al., 1995).

Prolonged exposure therapy involves systematic repeated confrontation with the traumatic memories (imaginal exposure) and with avoided trauma‐related situations (in vivo exposure). According to emotional processing theory, these exposures present patients with information that disconfirms the pathological elements of the pathological emotional structure, thereby ameliorating PTSD symptoms (Foa & Kozak, 1986). Emotional processing theory posits that effective treatment involves (1) activation of the memory structure (i.e. going to crowded places, talking about the event) and (2) the availability of corrective information, (i.e. ‘the crowed place is actually safe’ and ‘I can talk about the trauma without falling apart’). During the activation of the memory structure, an optimal level of activation is necessary, too little activation (emotional under engagement) and too much activation (emotional over engagement) can impede the efficacy of the treatment (Foa & Kozak, 1986).

The PE treatment protocol was developed by Foa and colleagues (Foa et al., 1991). PE is a short‐term treatment consisting of 8–15 sessions that are 90–120 min in length, and generally in an individual format. The goal of the treatment program is to ameliorate the severity of chronic PTSD symptoms. Core components of therapy include (1) relaxation training through breathing; (2) psychoeducation regarding treatment rationale and common reactions to trauma, (3) In Vivo Exposure: gradual exposure to avoided trauma‐related but objectively safe situations; (4) imaginal exposure: repeated recounting of the traumatic memory; and (5) processing erroneous cognitions related to the traumatic memory and processing negative emotions like fear, guilt, shame, anger and sadness. The components of the exposures are the central components of the treatment and these include generating a hierarchical list of trauma reminders, gradual exposure to them as homework between the sessions and the use of imaginal exposure within the sessions. In imaginal exposure, the patient is asked to revisit the traumatic memory and recount the traumatic memory for about 30 min over and over from the third session until one session before the last session. The patient is asked to recount the traumatic memory out loud, in the present tense and in the first person whilst closing his eyes. The patient is encouraged to describe the event in detail whilst describing the feelings, behaviours and thoughts that arose during the event and to be emotionally engage with his feelings. The narrative of the traumatic memory is recorded, and as homework the patient has to listen to the recording.

The second therapy assessed in the current study was that of Somatic Experiencing (SE). Somatic Experiencing is a body‐awareness approach to healing trauma developed by Peter Levine in the 1980s and based on the premise that rather than a pathology, PTSD is a ‘natural process gone awry’ (Levine & Fredrick, 1997, p. 6). According to the theory underlying SE practice, trauma occurs following the experiencing of potentially life threatening events when a person is strained beyond their ‘adaptational capacity’ to regulate arousal states (Levine & Fredrick, 1997). SE references animal observations times of attack demonstrating tonic immobility, manifested as a paralysis, one of global fixation or freeze, of survival energy. One factor indicative of a post trauma response in humans is when fear from instinctive, frozen defensive movements is unable to be discharged (Levine, 2010, p. 23). Treatment in SE is focused on accessing potential resources to reduce symptoms and restoring the body's natural capacity to regulate states of arousal, by discharging the energy that has accumulated in the body during overwhelming threat (Levine & Fredrick, 1997).

From this theoretical perspective, the goal of SE therapy is to heal the traumatic response by developing a capacity for tolerating extreme sensations and related emotions (Levine & Fredrick, 1997) enabling what is termed a ‘discharge of survival energy’ (Levine, 2010, p. 14). As opposed to cognitive based therapies, SE focuses on capacities for stress self‐regulation (Payne et al., 2015) and is not exclusively formulaic or a codified protocol, though it is built on an unfolding process that involves basic principles and building blocks (Levine, 2024). Treatment includes the use of specific, body based tools to work with instinctive defensive body patterns, either whilst working with specific conscious memory or non‐verbal sensations or experiences surrounding the event but not necessarily actively recalling or remembering the event (Levine, 2010). In SE the therapist uses the principle of titration, by which they introduce small levels of activation, to let the client gradually experience the traumatic event, without becoming overwhelmed, a stance which according to the therapy, prevents re‐traumatization (Levine, 2010). Despite claims of releasing stuck energy, enhancing interoception and connection to the body promoting physiological and emotional regulation (Levine & Fredrick, 1997) there is no rigorous research to evidence these claims. SE has been shown to reduce trauma symptoms in PTSD sufferers (Brom et al., 2017) and in a single session study (Parker et al., 2008) though mechanisms of change and non‐wait list comparisons have not been studied.

Whilst PE has a large evidence base, SE has few rigorous scientific studies and a limited evidence base including limited evidence on attrition. There is a need to determine whether SE is an appropriate and effective treatment for veterans with PTSD given the current success rates and levels of attrition across PTSD treatments. Additionally, given the differences in the two treatments studied and the current trend towards more somatic and mind–body treatments for trauma (Bisson & Olff, 2021), there is a need to delineate the central elements of the two treatments and ascertain whether their differentiation is warranted. The current study will attempt to describe the participant's experience and isolate elements which they felt were important for them in the therapy experience. Rather than comparing treatments, we will attempt to discover the central tenant of the therapeutic experience for participants using a descriptive phenomenological approach in order to focus the analysis on the essential essence of experience (Todres, 2005).

## METHOD

### Participants

**FIGURE 1:**
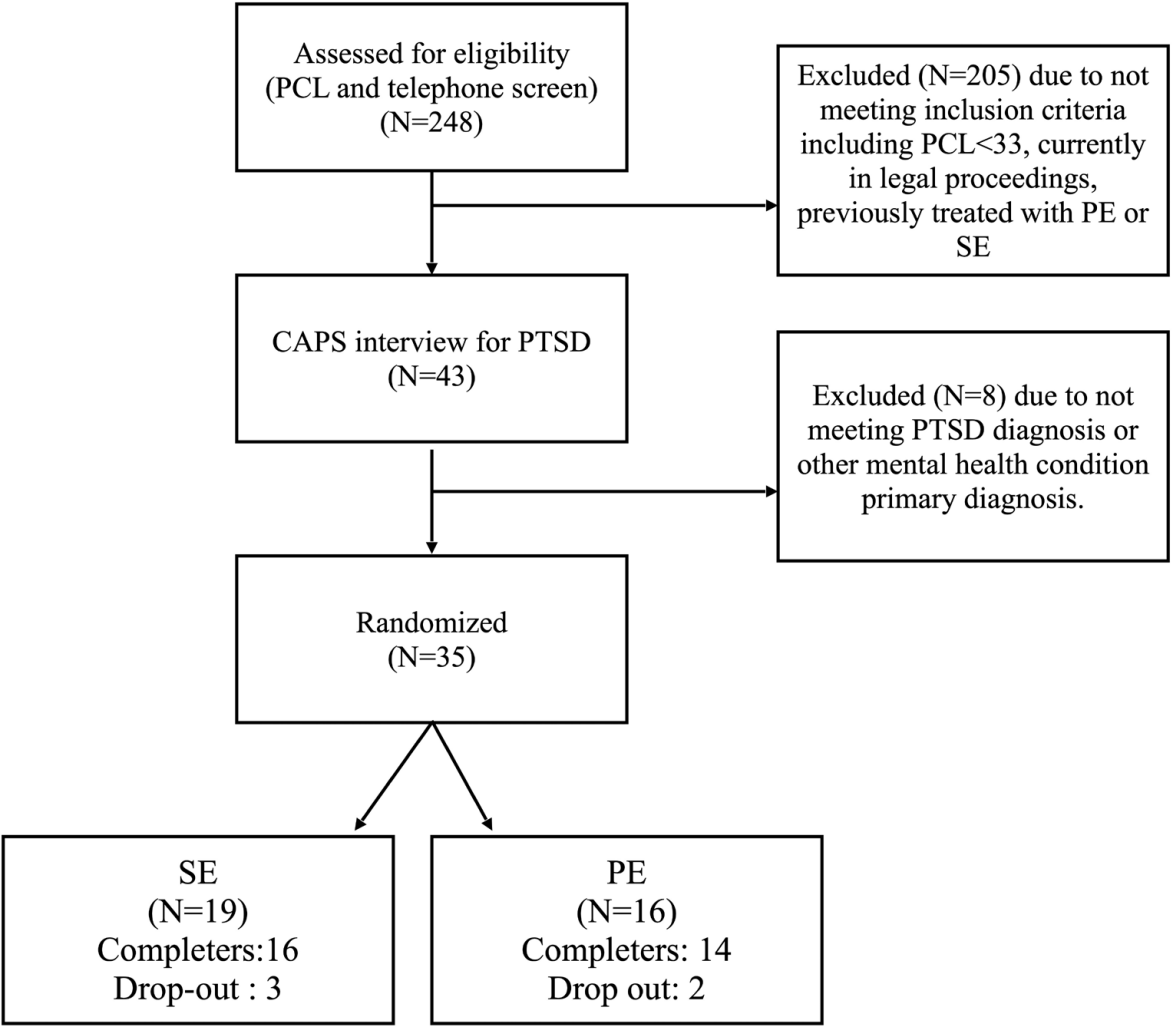
Recruitment and selection flow chart for RCT until midway point.

Thirty participants were recruited from a randomized controlled trial (RCT) into the effects of SE and PE on PTSD in veterans at the midway point (see Figure 1). Participants (26 male, 4 female), ranged from age 24–60 and had a mean beginning CAPS‐5 (Weathers et al., 2018) score of 32 (*SD* = 11.1). All participants met the DSM‐5 criteria for PTSD with an index trauma which was military related. Participants were all Jewish and Israeli former soldiers from the Israeli Defence Forces. Additional demographic and trauma‐related information can be seen in Data S1. Five participants (3 from SE and 2 from PE) dropped out of the study and did not have final interviews.

Study was approved by the Institutional Review Board (Helsinki committee) at Bar Ilan University and main RCT pre‐registered at the open science framework (https://osf.io/h8gmw). All participants gave informed consent to take part in the study and participation in the interview was optional.

### Therapy schedule

Participants were randomized to receive either SE which comprised of 21 sessions, each 50 min in length, or PE, 14 sessions (13 and an intake session), each 90 min in length (total therapy time for each therapy was 21 h). All therapies were conducted in the therapists' private clinics and all were licensed with additional specialization in either SE or PE. Therapists followed recognized protocols as outlined in the introduction (available upon request) and received supervision from an SE or PE expert throughout the trial. PE sessions were recorded according to PE protocol.

### Procedure

Participants were interviewed by the research coordinator (ShE) following completion of therapy. The interview was structured and all participants received the same order of questions.

### Data analysis

Analysis followed the principles of thematic analysis (Braun & Clarke, 2021) though was performed from a descriptive phenomenological approach (Todres, 2005). The analysis of responses consisted of four stages. During the first stage, the two lead researchers AHG and ShE read all the blinded transcripts and labelled potential codes. These codes were concurrently entered into the master document thus each researcher was influenced by the codes of the other and similar codes were noted and discussed. During this phase it was clear that despite treatment type being blinded, there were clear indications in the text and that in many cases both researchers could identify which treatment had occurred (PE or SE). During the second stage, the two lead researchers discussed common codes and began to identify potential themes. Potential themes were discussed with an external qualitative advisor, AB, and then decided upon by AHG and ShE. In the third phase, codes were classified as belonging to themes and discrepancies discussed. In the fourth stage, upon classification, interviews were unblinded and the final spreadsheet included with treatment assignment alongside quotes, codes and themes. Differences between treatments were discussed. Findings were assessed with DB, NN (PE supervisor), KL (SE supervisor) and CL (SE therapist recruiter) with all associate researchers reviewing final coding, classification and final thematic analysis.

As defined above, the analysis process was based on several layers of multiple coding, which allowed for the reliability and trustworthiness of our results. Multiple coding entails that interviews are analysed individually by two or more researchers that meet on a regular basis to discuss agreements, disagreements and alternative interpretations to the interviews, thus allowing for an analysis that is multifaceted and unbiased. Multiple coding is acknowledged as a vehicle towards qualitative research trustworthiness and reliability (see, Mays & Pope, 1995). In this regard, it is important to note that multiple coding is not the same as inter‐rater reliability. Whilst the latter is aimed at agreement the former allows for disagreement and debate, in the purpose of exploring all possible interpretations in the pursuit of the best and most reliable analysis (see, Barbour, 2001, p. 1116).

Since all the authors had clinical backgrounds, and some had a strong professional predisposition towards SE or PE, we engaged in introspective reflexivity throughout the analysis process (Patnaik, 2013). Specifically, we took steps to ensure that the authors' professional perspectives did not limit their ability to gain new insights into SE and PE. These steps included: (a) Ensuring that all researchers—distinguished professionals in fields such as SE, PE, psychodynamic and constructivist psychotherapy, and integrative CBT approaches to trauma—assessed the findings and offered alternative interpretations. This theoretical triangulation (Stahl & King, 2020) allowed for multiple theoretical perspectives to inform both the analysis and the final results. (b) During the final stages of analysis, we facilitated face‐to‐face discussions via zoom meetings amongst all the authors, based on a final draft of the results. These discussions focused mainly on gaps between the researchers' initial perspectives on SE and PE and the new insights that emerged from the study. This process ensured that professional biases did not limit the consideration of new perspectives.

## RESULTS

Results are presented as three overarching themes, experience of change, experience of the relationship and experience of the process, which are explored in relation to the two therapy modalities.

## EXPERIENCE OF CHANGE

### Somatic experiencing: changes from within to movement outside

Participants in the SE group perceived change in terms of a growing awareness of their physiological and mental responses to traumatic and other life experiences. These changes from within allowed for a ‘movement’ outside, in real‐life. This awareness was described through three different concepts: Learning about what happens to the body, learning to cope with the symptoms and reconnecting and rediscovering the body. There was a central focus of the body which penetrated all these concepts.

#### Learning about what happens to the body

Learning was described as; ‘learning what happens in my mind and body in terms of knowledge, that I know what is happening to me and physically take note of how I am feeling in my body’ (participant 20).

This learning was experienced, not only as a cognitive process, but also as reconnecting to the body; ‘I always felt there was a cut between my body and my mind. He taught me that, that's what I am feeling, that if you don't pay attention to your body, it is harder’ (participant 18).

#### Learning to cope

Whilst participants referred to symptoms, it was in reference to coping with them, rather than a disappearance. The majority of participants perceived change as coping with or gaining control over their symptoms; ‘it was just to talk in the past, without feeling. Suddenly you feel this thing, more in your body….it helped me feel more in control, to feel it a little less…’ (participant 18). By experiencing control over their symptoms participants were able to shift their emphasis from symptom reduction to self‐regulation, as one of the participants explained:

I don't think it helped with the symptoms themselves, that is to say, they didn't go down in themselves, like sleep or hyperarousal, rather it was how to deal with the symptoms…to give more space to the things instead of jumping to why it is like this and attacking the problem. (participant 2)

#### Reconnecting to and rediscovering the body

Participants emphasized the body when describing their experience in SE, ‘the use of the body, it was the non‐verbal things which caused the movement’ (participant 10) and that reconnection with the body and the bodily response to trauma brought about change, ‘Something about breathing and to connect to the moment, to what you are going through… the ability to calm myself (changed)’ (participant 4). This ability was also reflected in a sense that participants received ‘tools’; ‘For example, the tools of going in and out is something that I am taking with me’ (Participant 26), which were useful beyond the therapy experience; ‘all the tools feel like resources which you can grab from them at a specific moment what is correct’ (participant 22).

### Prolonged exposure: reducing symptoms, internal change and coping

Amongst the PE participants, change was perceived as a reduction in symptoms, an internal change and coping. These elements of change were described both as a process of learning and as an outcome and heavily featured the physiological or bodily experience.

#### Symptom change

Participants described the change following treatment in terms of the reduction of diagnostic symptom categories. These were described both in relation to specific PTSD symptoms and also in the symptoms experienced during the re‐experiencing of the trauma. Symptoms were generally defined referring to a symptom from the DSM‐5 diagnostic criterion ‘I suddenly notice that the last week went ok, I encountered a situation where two weeks ago I would have been with a very short fuse and now, suddenly, I'm more relaxed’. (participant 9) but also more generally, ‘I feel alive and breathing’. (participant 7). They were described in relation to the weekly checklists which were discussed with the therapist, ‘it seems from the questionnaires that she (the therapist) gave me, that there was a change both in behavior and in symptoms, that I see we made a difference to them’ (participant 182) and this was related to their experience outside of the therapy room:

I see the numbers and see that they are going down, of the questionnaires, I once said six months ago I wanted to commit suicide. Today it is not in the lexicon. Significant change. As of today, a week after graduation, I definitely see change and improvement. (participant 17)

Symptoms were described in relation to the exposure to the traumatic experience during the therapy session:

In the first exposures, my body reacted quite powerfully, My legs were shaking, I felt a sense of suffocation and the contraction of my body and my breathing was so flat. And as we progressed with the process, the exposures became easier in this respect that in the end there were no longer any tremors and in general, it was no longer difficult to talk about it and it was even exhausting to talk about it. The concerns pretty much disappeared, it was easier to talk about these things. (participant 3)

#### Internal change

In addition to the reduction in symptoms, participants underwent a process of what they described as internal change, that is, a change which was not quantifiable or referring to a change in stance or mindset. ‘It affected something internally. I don't know but if you ask those around you how much {name} has changed, they will tell you wow {name} has changed, but something internal has changed a lot for me’. (participant 5) and at other times expressed in relation to understanding and acceptance. Participant 19 referred to his nightmares, ‘It didn't change my nightmares but I could look at them differently, with a greater understanding’ and others ascribed this internal change to their emotional experiences: ‘People were always talking to me about guilt but I didn't feel guilty. It became clear to be that there was a specific amount of guilt. It became more focused, deep down, on what I actually felt guilty about’ (participant 3).

#### Coping

The result of both the reduction of symptoms and an internal change was an increased sense of coping. Participants described a transference of therapy gains to the outside world, ‘I feel that I have more awareness and more ability to deal with the world. I know there will be more triggers and more periods and something in me is a little less frightened than that’ (participant 24). Coping was described as a process of understanding and acceptance, ‘I got a lot of understanding about what is happening to me. To understand that I have the PTSD and anxiety and when I have anxiety in my body…I learnt to put my finger on when my body is bubbling and to learn to accept it’ (participant 30) and also described passively, ‘I suddenly notice that the last week went ok, I encountered a situation where two weeks ago I would have been with a very short fuse and now, suddenly, I'm more relaxed’. (participant 9).

## EXPERIENCE OF THE THERAPEUTIC RELATIONSHIP

### Somatic experiencing: leading, walking side‐by‐side

For participants in the SE group, they reflected on the therapeutic relationship as a central tenet in the experience of the therapy. This relationship was described as that of a caring person who saw the participant and walked alongside the participant, at the participants pace. Concurrently, the therapy experience was described as led by an expert. Walking alongside did not appear to come at the expense of a focused therapy experience, rather paced and gentle.

The caring and excellence of the therapist predominated in participants' descriptions of the relationship, as long as it was accompanied by a sense of genuine connection:

The therapist…was one of the most influential women that I have met in my life… It's a great fortune that there are therapists like this, and that injured people like us can get help. In the end it is all about a connection between two people. (participant 1)

Together, participants felt they were able to traverse a therapeutic journey. This journey, however, was possible, as long as there was a therapeutic joining: ‘[the therapist] was not normal, I've been in therapies in the past and she was the first who really was able to get, together with me, to a … of process of deep change’, (participant 10).

The ‘right’ pace was perceived by participants as one of the most important traits of the therapist, as it allowed for a feeling of attentiveness to the client along with a sense of authority to gently lead. Participants described feeling ‘embraced, contained and understood’ (participant 20). Alongside the caring nature of the therapist, participants described a sense of security and control projected by therapist, ‘doing things at the right pace…. to feel that she was in control’ (participant 26). This sense of security was described as coming from the therapist ‘(in the past) there would be an explosion and then I would quit and suddenly with him I felt a sense of security’ and as developing within the participant ‘(the therapist) helped me to feel more in control’ (participant 18).

The alternation of therapy being led by the participants' needs and at the same time led by an expert was a recurrent theme. On the one hand there was an understanding that ‘the therapist was very attentive to my needs’ (participant 30) and on the other hand, that the therapist was being a lead by a focused expert ‘(the therapist) was focused and amiable’ (participant 25). One of the participants explained about this concurrence: ‘(the therapist) was prepared for every meeting….it gives you faith that what you are going to go through will help’ (participant 23). Whilst the majority of participants did not elucidate what this focus was on, one participant described:

I came with baggage and a needed to let it out, beyond the military traumas, so perhaps I came and I was all over the place and shared a lot but [therapist] wanted to always bring me back to the military trauma. (participant 20)

### Prolonged exposure: a recipe for success prepared with warmth and care

In the PE group, participants referred to a sense of order or focused therapy which was reflected in descriptions of the therapy and the therapist. Whilst the focused therapy was seen as central to the therapy experience, this focus was combined with descriptions of the warm and sensitive nature of the therapist which enabled the participant and therapist to walk hand‐in‐hand.

Participants described the trauma focus of the therapy as central to the experience, ‘it was clear to me that the significant trauma was the focus and for that, the therapy was accurate’ (participant 21). Knowing that the therapy would be focused and according to a protocol aided a sense of security which aided adherence to therapy:

One of the things which strengthened me, was that I received from (the therapist) the treatment workbook that highlights that regression is predicted to be part it and that it is part of the therapy. That gave me the strength during the difficult times…. it was pleasant in her therapy room. (participant 182)

The organization of the therapy, was described as ‘a recipe’ which ‘created order, there are steps that you prepare for mentally’ (participant 24). In this case, the participant described opening the therapy manual and looking at upcoming sessions in order to be prepared.

The focus of the therapy was seen as novel to this therapy, with one participant comparing to previous therapies, ‘to open the trauma and in previous therapies they (the therapists) had avoided that….it was more aggressive and deeper’ (participant 7). The focused depth and aggressiveness was perhaps enabled by the complementary nature of the therapists: ‘[therapist] walked with me hand in hand’. (participant 24).

This combination of excellence and focused nature of the therapist, described in terms of ‘an incredible level of accuracy’ (participant 5) and ‘to the point’ (participant 17) was complemented by a high level of sensitivity and caring:

I don't know if this is part of the concept of PE or that it was [therapist]. She loves her work and you feel it…I really appreciate her. When you feel someone is there for you and gives from himself, and he cares, that's an important combination for treatment success. (participant 17)

Participants described feeling comfortable in the presence of the therapist, that they were ‘sensitive and not just technical’ (participant 17) and ‘without judgement’ (participant 30).

## THE EXPERIENCE OF PROCESS

### Somatic experiencing: Learning an unspoken language

For participants in the SE group, the process appeared to hinge on learning an unspoken language, that of the body. The process was a gradual one but involved a critical moment of internalizing both the unspoken language of the body and that of acceptance rather than an overt expression of change.

Learning the unspoken language predominated in participants' description of the therapeutic process. A central component of this ‘language’ was often not speaking the regular ‘other’ language that participants are used to talk, as one of the participants described:

… the fourth session I think, I remember that after the sessions you fill in a questionnaire and it asks you did you talk about things that felt right to you, and in this method you don't talk a lot and I realized that it was *that* which was meaningful. (participant 1)

Talking less and working more with the body was not immediately clear to participants, ‘All this talking less and more calming, I've never been through something similar in my life, but slowly, slowly, I gave it a chance and (then) you understand’ (participant 8).

Difficulty working with the language of the therapy took time to get to grips with, ‘It took (therapist) a lot of time to get me used to this language but the moment that I succeeded, it was really useful to me, and I understood what was unique about this therapy’. (participant 10). The language of the therapy involved using the body but also involved reframing how they described their purpose in therapy. ‘When I requested this therapy it was with the thought of like putting tipex (white‐out/eraser) on the event, and at the end of the therapy I realized that there was something inside of me that didn't want to wipe it out, rather simply learn to live with it and I think those are the tools I got’ (participant 4).

Learning to live with symptoms appeared to be a crucial turning point in participants' therapy, as they learned to see and describe the symptoms in a nuanced language of the body that enables the awareness of, and reaction to, change: ‘the symptoms are there all the time. There are times of high intensity and times of low intensity. I learnt to breathe with them’ (participant 1). The ongoing process was repeated and highlighted: ‘it is work for a lifetime, it doesn't disappear’ (participant 22).

### Prolonged exposure: Tolerating the climb to conquer the peek

For participants in the PE group, taking part in ‘exposures’ was central to the therapy process. The therapy process was described as comprised of ups and downs which eventually stabilized. This stabilization appeared to occur towards the end of the therapy, as a peek which was conquered, and thereafter little change occurred, rather, gains were stabilized.

Descriptions of PE therapy predominantly alluded to the exposures as central to the treatment. Following initial psychoeducation, ‘we began with exposures that touch upon the traumatic event that I underwent, there it was all kinds of vagueness and it was a sort of worry to talk about it’. (participant 3). In addition to directly referring to exposures, participants described them as repetitive retelling of the traumatic events:

It was very focused on repeating the same events, sometimes it felt technical… and maybe you miss essential things and on the other hand it completely took the load off these events… it's a method that doesn't give up on you and maybe that's what's needed… and maybe that's what's difficult for me, that it touched at the point itself. (participant 15)

The exposures were experienced both during sessions and also in confronting avoidance outside of the therapy room, ‘At first it was really hard for me. It was difficult to leave the house and also (to go) the therapy session itself. Little by little it got better, I felt more comfortable coming…also thanks to the exposures’ (participant 13).

The experience of being in therapy for many participants was difficult and frequently described as a process with highs and lows alternating:

The process was an emotional rollercoaster. There are times when you are ok and there are two or three days after the therapy that are a complete down. It is completely an up and down, and towards the end I felt routine and improvement. (participant 17)

The rollercoaster was also described as waves of emotional experiences, ‘It's an emotional rollercoaster…everything in itself is a process, (the therapist) described it as a wave, and I agree with her, up and down and again an up and so forth’ (participant 30). These ups and downs made the process difficult, ‘It is not an easy treatment. There were weeks that really broke me down, there were difficult weeks, there were weeks in which I could feel the help, in the last two meetings I felt good’ (participant 24). Difficult sessions even led participants to consider ending the treatment, ‘There were many times that I wanted to end but with the last sessions, my state improved a lot, I even managed to conquer the peak’ (participant 21).

Sessions were described as accompanied by ‘a difficult day both before and after (the session)’ (participant 9), ‘After each session you have two days of being crushed, I smoke and (have) stress and anxiety, difficult days…. I wanted to quit not once or twice, and not for me, for my family’ (participant 21). Conquering the peak, as described above, appeared to correlate with the end of the difficulties experienced prior to and following sessions. When the ‘peek’ was conquered, the difficult periods of increased symptoms were relieved, ‘In the last weeks, three weeks, there really was a reduction in symptoms…. there was a relief in the deterioration that had been during the previous two months’ (participant 9).

Participants described a period of stabilization towards the end of the treatment which was reflected by a retention of gains, ‘In the 2‐3 last sessions I was in the same state as now, that is a relative sense of finishing’. (participant 17) and a sense of the exhaustion of treatment benefits, ‘From the twelfth session it felt a little like (I) exhausted (the treatment) and improvement’. (participant 17).

## DISCUSSION

The current study explored participant's experiences in two psychotherapies for PTSD. Each therapy is based on different theories of action, both in terms of the foundation of a PTSD response and in regard to the mechanisms of change of the treatment. Whilst the difference in theoretical basis is clear (as outlined previously), the experience of the participants in both therapies highlighted overarching similarities, peppered with nuanced differences. These were reflected in the experience of the therapeutic relationship, the experience of the process and the experience of change. Identifying and acknowledging these nuanced differences strengthens the claim that qualitative research focusing on the lived experience of PTSD patients might complement the ‘bigger’ picture provided by quantitative research with high resolution information (Doran et al., 2021; Hundt et al., 2020; Meis et al., 2022; Sherrill et al., 2022).

Indeed, the above‐mentioned differences, as nuanced as they might be, reinforce that differentiating between patient needs, different levels, symptoms structures and severities of PTSD might assist in achieving better intervention results (see, Burback et al., 2023; McFarlane et al., 2017) based on selecting the most effective therapy for each case. In this regard, understanding the differences between different kinds of therapies in terms of the user‐experience, might counter the poor rates of remission (Forbes et al., 2019) and/or lower the rates of attrition (Schnurr et al., 2022) in PTSD therapies. Specifically, although it has been acknowledged that SE provides good clinical results, there is need for more scientific evidence (see, Almeida et al., 2020), and though PE has been proven to have lasting significant results (Schnurr et al., 2022), specifically in US samples, there are high levels of attrition. It might be the case that addressing the nuanced differences between the two interventions, might assist in deciding which therapy is suitable for whom and delineating the key experienced methods of change thus providing greater pre‐treatment preparation. These understandings might also serve to enhance the efficacy of new methods for the treatment of PTSD that integrate elements of these PE and/or SE (e.g. Bisson & Olff, 2021; Burback et al., 2023).

In both therapies the role of an expert and focused therapist was highlighted as central to the experience of therapy. In SE, the importance of traversing a journey together was highlighted, reflected in the discussion of pace, a sense of security and a sense of focus. The use of the word gentle or gently is applied in Levine's method to varied elements of the therapeutic process and is highlighted in many of Levin's writings (see, for instance Levine & Fredrick, 1997). A ‘gentle’ process, in the SE literature, is manifested through a gradual exposure that begins by slowly acknowledging sensatory experiences (or traces of traumatic arousal), focusing on one element of the experience at a time. The management of heightened arousal then also stems from a gentle and compassionate approach, monitoring closely to prevent overwhelming recollection of traumatic memories (Levine & Fredrick, 1997). Acknowledging that slow pace and ‘gentleness’ are essential elements of SE, as our research participants highlighted, might become a consideration for therapists contemplating weather SE is suitable for their clients.

Additionally, it is perhaps this close monitoring in SE which is sensed by participants to indicate attentiveness and an experience of walking side‐by‐side, which is related to the development of a sense of security and control both emanating from the therapist and from within. The experience of security and control can be framed as a corrective experience in contradiction to traumatic experiences characterized by fear, a sense of being overwhelmed and helplessness (for a discussion on the importance of corrective experience in CBT based interventions, see, Hayes et al., 2012).The creation of a safe therapeutic space, the maintenance of a close and attuned relationship and the importance of the corrective nature of the relationship, which can be identified in participants' experiences, is in coherence with the centrality of connection within the SE therapeutic approach. Indeed in many current approaches to trauma conceptualizations (Herman, 2023; Mikulincer et al., 2015; Sharma et al., 2020), and SE in particular, trauma is conceived as the absence of an ‘empathetic, mutually connected, witness’ in addition to the essence of the traumatic event (Levine, 2024, p. 20). The current study demonstrates how such witnessing becomes central in the particularity of the SE experience.

In the PE group, the caring and sensitive therapeutic relationship was also highlighted in participants' experience of the therapy. Similar to those in the SE group, participants described the therapist as walking hand in hand through the therapeutic journey but more predominate in the PE group, this account was accompanied by descriptions of the accuracy and focus, of the therapy, specifically, conforming to a ‘recipe book’. This reflected the manualized protocol in which each session has a clear and structured agenda. Despite the reported tensions described in the literature between relationships, therapeutic alliance and adherence to protocol, (Barak et al., 2014; Chen et al., 2020), the adherence to protocol (fidelity) did not appear to come at the expense of the therapeutic alliance. Nonetheless, the protocoled nature of the therapy was clearly highlighted as central to the therapeutic experience and thus motivation and expectation of treatment style appears to be a consideration for PE participation. In the current study, this ‘recipe book’ experience was in coherence with the therapy ‘sign‐posting’ encouraged in supervision sessions coupled with the importance of forging a strong therapeutic relationship highlighted in PE training. This is framed as both to help implement in vivo and imaginal exposure and also to help the patient to be emotionally engaged in the traumatic memory (Foa & Kozak, 1986).

Indeed, the induction of the sense of safety and caring in PE therapy was evident in participants' accounts and was acknowledged by them as reducing fear of the traumatic memory, allowing them to engage in processing and sharing of difficult content as well as in completing homework assignments between sessions (Kazantzis et al., 2000). It is important to note that in particular, the emphasis on a caring stance may be reflected in the cultural setting of the therapy with the Israeli therapists experiencing greater affiliation with patients given shared environmental and cultural factors associated with ongoing continuous stress (Ennis et al., 2020, 2021).

Participants in both groups reflected on the expertise and adherence to the specific methodology. Whether described as confidence in the expertise of the therapist, the protocoled nature of the therapy or the specific language, the distinction of the method was important to participants. In recent years there has been a move to integrative therapy, one which enables therapists to utilize multiple techniques led by patient need (Horesh & Lahav, 2024; Zarbo et al., 2016). Based on the current participants' experiences, there is an indication, specifically in trauma therapy, that the focus and expertise identified as adherence to a method, was of value to the success of the treatment. Thus, it is important to consider whether integration, as suggested and implemented in many areas of PTSD treatments (Bisson & Olff, 2021; Bongaerts et al., 2022; Lahad et al., 2016; Voorendonk et al., 2020; Wright et al., 2022) comes at the expense of specialization. Based on the current findings, and whilst accepting the claim that the therapeutic alliance is central to treatment success, regardless of the kind of treatment (Steil et al., 2023), it is noteworthy that the stance of an ‘expert’ in a particular method, is important to clients and seems central to their ability to forge alliance and rapport with their therapist.

In addition to an emphasis on relational characteristics, participants reflected on their therapeutic journeys or processes. For SE participants, the process of change and eventual experienced results centred around learning a new language, predominantly unspoken, of the bodily response to experiences. The SE literature refers heavily to the learning of a new language, facilitated by interoception, often termed the ‘felt‐sense’, through which one can ‘experience well‐being, peace and connectedness’ (Levine, 2010, p. 72) by learning to connect and interact with the body's internal state. The language is stratified ‘from physical sensations to feelings, perceptions and finally thoughts’ (Levine, 2010, p. 139). The current findings have the potential to add to the existing SE literature, offering new insights about the lived experience of learning this new language, mainly the importance of learning to appreciate the value of listening in processing traumatic experiences and rather than just use the bodily experience as a language to express trauma, learning to notice the bodily experience by way of expressing trauma, enables a stance of acceptance and noticing rather than These insights might assist SE practitioners in attuning to their clients as they learn the SE language.

The experience of the body as central to reflection on change was predominant not only in the SE experience, but also in the experiences of participants in the PE group. The monitoring of bodily responses is central to the emotional processing theory, which underlines PE (Foa & McLean, 2016). In PE there is not an emphasis on the body, rather it is used to define subjective units of distress, a method of monitoring activation. This enables the noticing of habituation and of distress reduction, an indicator of successful treatment outcome (Rauch et al., 2004). During the imaginal exposure the levels of distress experienced by the patient are taken to be a sign of his degree of emotional engagement in the therapy which is necessary for successful outcomes. The therapist is taught to pay attention to the patient's facial expressions and body movements (moving legs, restlessness) in order to increase emotional engagement if there are little signs of physical arousal (under engagement) or reduce it if the patient is in great distress and overwhelmed (over engagement). Given the centrality of body in the clients' accounts it might be suggested that a more holistic body awareness could be emphasized in PE to help patients who are under engaged (telling the narrative without emotion) to lever the influence of the treatment. Such integration has only been scarcely explored in the literature, specifically in relation to an adaptation of CBT with elements including those of SE in order to produce the SEE FAR approach (Lahad et al., 2016). The current results warrant the exploration of this integration which could further benefit client experience as well as treatment outcome beyond the reduction of PTSD symptoms.

Treatment outcome was referred to when participants described their experience of the therapeutic process. The pace of the process of each therapy was noticeably different for the two groups' participants. SE participants reported a process of acceptance and integration into their life and PE participants described a difficult process and then a noticeable change, most succinctly described as the conquering of a peak. This marked, or in some cases, sudden, change followed by stabilization was not evident in the SE group. Indeed, the PE protocol, based on Foa & Kozak's theory of PTSD (Foa & Kozak, 1986) is focused on symptom reduction as the goal of treatment (Foa et al., 1991) through, what was described by our research participants as, a challenging and repetitive process that enabled changes in internal narratives and cognitions (Foa et al., 1999). It appears that for participants' in the SE group, symptom change was less prominent in their experience of the therapy, rather an overall change in how dysregulation and emotional discomfort is addressed. In this regard, this has implications for suitability for treatment when symptoms are vague or not fully meeting PTSD criteria. For potential users of PE therapy, it appears that clear symptom profiles and willingness to partake in the challenging therapy may be conditions for participation. This should be assessed prior to treatment assignment whilst highlighting that taking the challenge could be profoundly worthwhile to them. In this regard, it is important to note that the emphasis on the challenging nature of PE therapy and a sense of not giving up is perhaps particularly relevant for this veteran population group. Conquering and overcoming are key parts of a ‘warrior’ identity which can be displaced when PTSD debilitates (Hooyer et al., 2020). The learnt coping developed though repeated exposure and *not giving up*, as described by participants may increase the level of self‐competence felt when overcoming difficulties and adversity. On the other hand, the more gentle approach offered by SE and the reduced focus on specific symptom reduction, may make SE a more appropriate therapy for those who's PTSD symptoms are less clearly defined or perhaps do not fully conform to PTSD clinical cut‐offs, though this was not explored in the current study.

The current study was limited by a number of factors. Primarily, data collection begun in response to the richness of participant feedback following completion of therapy. Future research would benefit from a dedicated qualitative research team from the beginning of study conception to enable a potentially richer dater set to expand on current theoretical underpinnings. Additionally, as mentioned above, there appear to be nuances intrinsic in the Israeli therapy style and population though this has not yet been studied, neither in protocoled or non‐protocoled psychotherapies. These differences should be explored and their findings used to influence extrapolation to a cross‐cultural population. Additionally, in future studies, if these limitations are addressed, there may be a greater opportunity for differentiation and delineation of different treatment pathways, specifically between the waves of somatic and cognitive treatments (Bisson & Olff, 2021).

### Conclusion

In conclusion, the current study highlighted the commonalities and differences between SE and PE as trauma therapies. Our results warrant some directions for further research. Mainly, in our study, both therapies were well received and the compassionate and caring nature of therapists in both groups was clearly emphasized. In order to determine whether this is a cultural‐based anomaly or inherent in SE and PE provision cross‐culturally, future research should compare current findings with a wider sample. Additionally, specifically relevant in the current climate of integrative therapeutic work, the specialization and clear therapeutic modality and language of the therapies in the current study were valued by participants. Future research should compare the experience of participants with therapists on various levels of protocol adherence.

## AUTHOR CONTRIBUTIONS

**Anna Harwood‐Gross**: Conceptualization; Investigation; Writing‐original draft; Methodology; Writing‐review & editing; Formal analysis; Data curation; Supervision. **Shir Elias**: Writing‐original draft; Formal analysis; Project administration; Investigation; Data curation. **Karen Lerner**: Writing‐review & editing; Supervision. **Nitsa Nacasch**: Writing‐review & editing; Supervision. **Cathy Lawi**: Writing‐review & editing; Funding acquisition. **Danny Brom**: Formal analysis; Funding acquisition; Writing‐review & editing. **Adi Barak**: Formal analysis; Supervision; Writing‐review & editing; Writing‐original draft; Methodology; Validation; Data curation.

## CONFLICT OF INTEREST STATEMENT

The lead authors (AHG and SE) have no conflict of interest. N.N receives payment for training PE therapists and K.L receives payment for training SE therapists. CL is the CEO of emotionaid an organization disseminating SE related therapy and was involved in recruiting therapists for the current study.

## Supporting information


Data S1.

## Data Availability

Study was pre‐registered at OSF and we can provide an anonymized excel of quotes and allocation upon request.
