# Supplementary material for: Veterans' experiences of somatic experiencing and prolonged exposure therapies for post‐traumatic stress disorder: A qualitative analysis
Source: Psychol Psychother. 2025 Jan 14;98(1):175–92. doi: 10.1111/papt.12570 (PMC11823315; doi:10.1111/papt.12570)
Supplement: Supplementary file 1 — Data S1. [file PAPT-98-175-s001.docx]

**Table 1**

*CAPS-5 scores and demographic information for all participants*

| Participant | Gender | Age | CAPS-5 pre | CAPS-5 post | Group |
| --- | --- | --- | --- | --- | --- |
| 1 | Male | 33 | 49 | 11 | SE |
| 2 | Male | 29 | 28 | 37 | SE |
| 3 | Male | 39 | 37 | - | PE |
| 4 | Male | 38 | 26 | 4 | SE |
| 5 | Male | 40 | 32 | 20 | PE |
| 6 | Male | 29 | 37 | 15 | SE |
| 7 | Male | 35 | 30 | 3 | PE |
| 8 | Male | 30 | 49 | 13 | SE |
| 9 | Male | 35 | 17 | 20 | PE |
| 10 | Male | 40 | 37 | 4 | SE |
| 11 | Male | 30 | 38 | 45 | PE |
| 12 | Male | 27 | 47 | 47 | SE |
| 13 | female | 24 | 35 | 1 | PE |
| 14 | Male | 30 | 39 | 26 | PE |
| 15 | Male | 42 | 37 | 14 | PE |
| 16 | Male | 28 | 38 | 39 | SE |
| 17 | Male | 32 | 33 | 2 | PE |
| 18 | female | 27 | 30 | 19 | SE |
| 19 | Male | 60 | 23 | 33 | SE |
| 20 | Male | 25 | 24 | 14 | SE |
| 21 | Male | 34 | 36 | 9 | PE |
| 22 | Male | 35 | 36 | 27 | SE |
| 23 | Male | 51 | 32 | 3 | SE |
| 24 | female | 26 | 46 | 12 | PE |
| 25 | Male | 43 | 40 | 26 | SE |
| 26 | Male | 23 | 26 | 17 | SE |
| 27 | Male | 38 | 22 | 10 | PE |
| 28 | Male | 48 | 50 | 27 | PE |
| 29 | Male | 29 | 18 | 11 | SE |
| 30 | female | 32 | 37 | 31 | PE |

Interview questions (in Hebrew):

1. איך היה החוויה שלך במפגשים השבועיים?
2. עכשיו שהגעת לסוף תהליך הטיפול, מה אתה חושב שהצרכים שלך? (אם לא ברור לו השאלה- לדוגמה האם הייתה רוצה המשך טיפול, טיפול אחר וכו)
3. כמה אתה מרגיש שהטיפול היה מתאים ומותאם לך ?
4. האם אתה מרגיש שהטיפול עזר לסימפטומים של הטראומה שהתמודדת\אתה מתמודד איתם?
5. האם אתה חושב שהטיפול השפיע על איכות החיים שלך ?
6. האם יש משהו נוסף שחשוב לך להגיד לנו לגבי הטיפול ?
7. האם יש משהו נוסף שחשוב לך להגיד לנו לגבי התהליך ?
